# The Biosynthesis of UDP-d-QuiNAc in Bacillus cereus ATCC 14579

**DOI:** 10.1371/journal.pone.0133790

**Published:** 2015-07-24

**Authors:** Soyoun Hwang, Avi Aronov, Maor Bar-Peled

**Affiliations:** 1 Complex Carbohydrate Research Center, University of Georgia, Athens, Georgia, United States of America; 2 Departments of Plant Biology, University of Georgia, Athens, Georgia, United States of America; ContraFect Corporation, UNITED STATES

## Abstract

N-acetylquinovosamine (2-acetamido-2,6-di-deoxy-d-glucose, QuiNAc) is a relatively rare amino sugar residue found in glycans of few pathogenic gram-negative bacteria where it can play a role in infection. However, little is known about QuiNAc-related polysaccharides in gram-positive bacteria. In a routine screen for bacillus glycan grown at defined medium, it was surprising to identify a QuiNAc residue in polysaccharides isolated from this gram-positive bacterium. To gain insight into the biosynthesis of these glycans, we report the identification of an operon in Bacillus cereus ATCC 14579 that contains two genes encoding activities not previously described in gram-positive bacteria. One gene encodes a UDP-N-acetylglucosamine C4,6-dehydratase, (abbreviated Pdeg) that converts UDP-GlcNAc to UDP-4-keto-4,6-d-deoxy-GlcNAc (UDP-2-acetamido-2,6-dideoxy-α-d-xylo-4-hexulose); and the second encodes a UDP-4-reductase (abbr. Preq) that converts UDP-4-keto-4,6-d-deoxy-GlcNAc to UDP-N-acetyl-quinovosamine in the presence of NADPH. Biochemical studies established that the sequential Pdeg and Preq reaction product is UDP-d-QuiNAc as determined by mass spectrometry and one- and two-dimensional NMR experiments. Also, unambiguous evidence for the conversions of the dehydratase product, UDP-α-d-4-keto-4,6-deoxy-GlcNAc, to UDP-α-d-QuiNAc was obtained using real-time ^1^H-NMR spectroscopy and mass spectrometry. The two genes overlap by 4 nucleotides and similar operon organization and identical gene sequences were also identified in a few other Bacillus species suggesting they may have similar roles in the lifecycle of this class of bacteria important to human health. Our results provide new information about the ability of Bacilli to form UDP-QuiNAc and will provide insight to evaluate their role in the biology of Bacillus.

## Introduction

UDP-N-acetylglucosamine, UDP-GlcNAc, is an activated nucleotide sugar found in all organisms and is essential to life. In humans, UDP-GlcNAc is a precursor for synthesis of glycoprotein, surface glycans, and it is also found in extracellular matrices of cells which contain a variety of different sugar polymers; in fungi and arthropod, it is required to form chitin for cell wall and exoskeleton structures, respectively, and in bacterium it is essential component for bacterial cell wall peptidoglycan assembly that is required to form the wall and protect the cells [1–4]. In most organisms, UDP-GlcNAc is not only a substrate for glycans but also an important precursor that is further metabolized to form many other nucleotide amino-sugars.

In the 1960’s, the late glycobiologist Nathan Sharon, identified and characterized several 2-amino-sugars derivatives of GlcNAc in Bacillus sp. including a D-fucosamine (2-amino-2,6-dideoxy-D-galactose), D-galactosamine, and the di-amino-sugar bacillosamine [5]. The 4-epimer of D-fucosamine is D-quinovosamine (2-amino-2,6-dideoxy-D-glucose, abbr. quinovosamine, QuiN). Some of the amino-group of 2-amino-sugars can be found in glycans in acetylated or de-acetylated forms. The acetylated form of QuiN is QuiNAc (2-acetoamido-2,6-dideoxy-D-glucose). QuiNAc was identified in several important gram-negative human bacterial pathogens including the lipopolysaccharide (LPS) from Brucella [6] and Legionella [7]. QuiNAc is also an amino-sugar component of the LPS structure of plant fixing bacterium Rhizobium [8]. In Rhizobium etli CE3, a QuiNAc residue is located in the outer core of O-chain polysaccharide (OPS) linked to a 3-deoxy-2-octulosonic acid (Kdo) residue in the inner core of the LPS [9]. Mutant strains that lack the QuiNAc [10–12] fail to infect the host cells, and a specific wreQ mutant strain where a QuiNAc residue is replaced by its 4-keto derivatives [13] gives rise to bacterium that infect the host cells but very slowly. These studies suggest that QuiNAc contained in LPS is an important residue that participates in a symbiotic relationship between Rhizobium etli CE3 and its plant host. In other gram negative pathogenic bacterium like Helicobacter pylori and Neisseria gonorrhoeae, a modified QuiNac sugar is found with additional acetamido group connected at C-4” forming a glycan with diNAcBac sugar residue (see [14]) for review of the biosynthetic route of UDP-diNAcBac).

**Fig 1:**
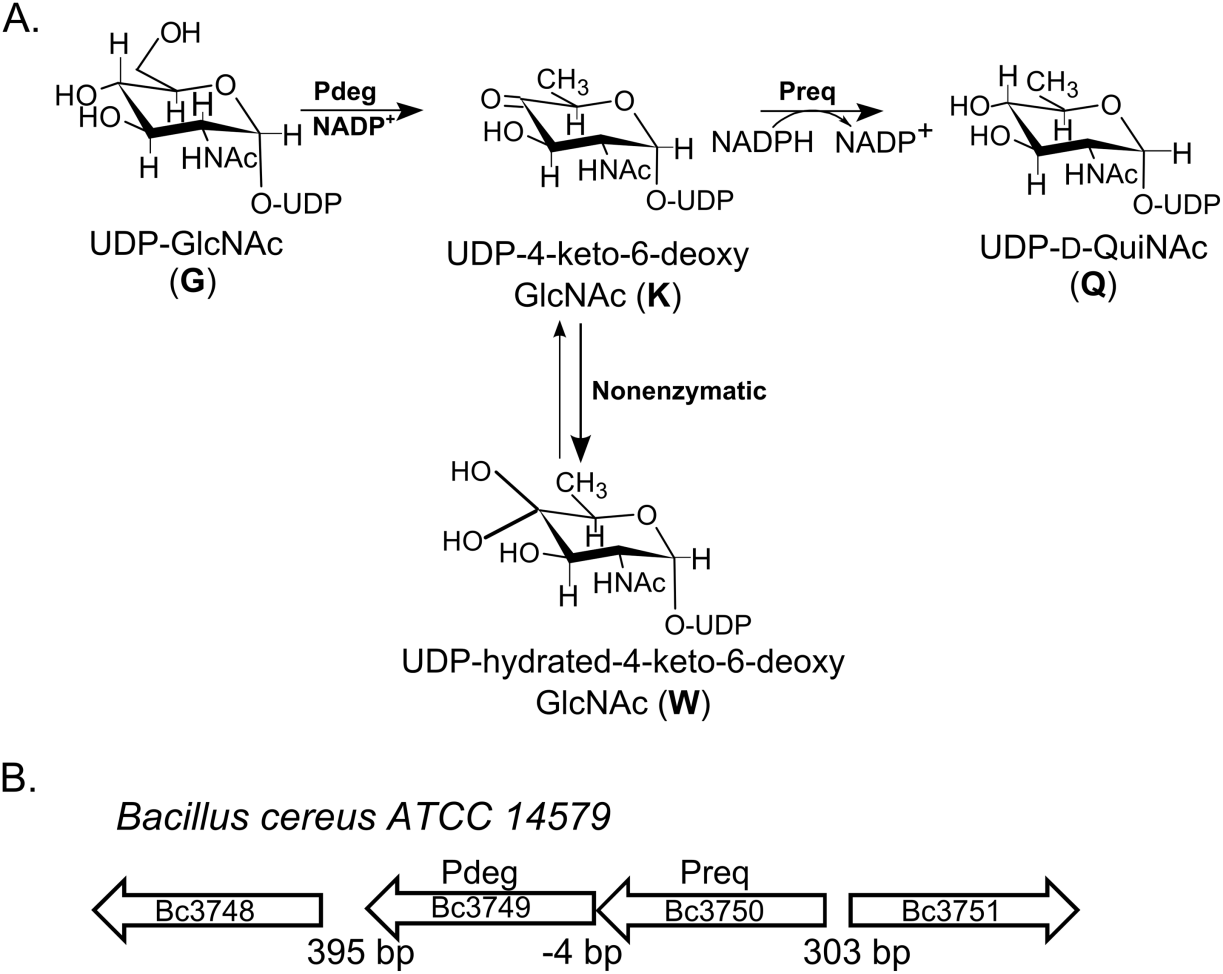
**A. A proposed pathway for the formation of UDP-QuiNAc in Bacillus cereus ATCC 14579.** The enzyme encoded by Bc3750, UDP-GlcNAc C4,6-dehydratase (Abbr. Pdeg), converts UDP-GlcNAc to UDP-4-keto-6-deoxy-GlcNAc. At steady state, the UDP-4-keto-sugar form (K) is converted non-enzymatically to a hydrated form W. The enzyme encoded by Bc3749 (Abbr. Preq) is a UDP-4-keto-sugar C4”-reductase and UDP-D-QuiNAc. **B. Organization of the two-genes operon and flanking regions in B. cereus ATCC 14579.**

By contrast, little is known about QuiNAc in gram-positive bacteria. In 1993, Ito et al [15] identified QuiN that was isolated from the cell walls of the alkaliphilic Bacillus sp. Y-25. QuiNAc, however, was not found in other Bacillus or any other gram-positive bacteria. Bacillus cereus is a food borne, spore-forming, and pathogenic bacterium, which is capable of motion by flagella. The bacterium is present in soil, dust, water, and plants (15–16). This Bacillus can also inhabit the intestinal tract of insects and mammals [16]. The bacterium is a facultative anaerobe and likely transiently present in insect cadavers and in decaying organic matter [17]. Bacillus is thus an attractive model to study the role of different polysaccharide structures that are made in response to different environments. Surprisingly, during routine analyses of polysaccharides derived from Bacillus sp, we detected an unusual amino-sugar and further analyses revealed this to be 2-acetamide-2,6-dideoxy-glucose, QuiNAc. However, little was known about the biochemical pathways and the corresponding genes involved in the formation of QuiN or its acetylated form QuiNAc in Bacillus. This prompted us to identify genes involved in the synthesis of QuiNAc-containing glycans. Here, we report the identification of a QuiNAc operon and the functional characterization of two enzymes that sequentially convert UDP-GlcNAc to UDP-QuiNAc (see Fig 1A) in Bacillus cereus ATCC 14579. Two bacillus enzymes encode UDP-GlcNAc-4,6-dehydratase and 4-reductase, which we named Pdeg and Preq. We used combined instrumentations with NMR spectroscopy and mass spectrometry to show that Pdeg converts UDP-D-GlcNAc to UDP-4-keto-6-deoxy-D-GlcNAc, and Preq immediately converts the 4-keto sugar to UDP-QuiNAc. Such enzyme activities have not previously been described in bacillus, and thus our data provides the basis for understanding the formation of QuiNAc-containing glycans by Bacillus and their roles.

## Materials and Method

### Bacterial strain and growth

Stock of wild type Bacillus cereus ATCC 14579 was stored in 30% glycerol at −80°C, streaked onto agar plate, and grown for 18 hours at 30°C. The medium (agar or liquid) used was Luria Bertani (LB per liter: 10 g tryptone, 5 g yeast extract, 10 g NaCl). Stock of E. coli strain DH10B (LifeTechnologies) was used for cloning, and strain Rosetta2(De3)pLysS (Novagen), was used to generate recombinant proteins.

### Cloning of Pdeg and Preq

A single colony of Bacillus cereus ATCC 14579 grown on LB-agar was suspended in 50 μl sterile water. The suspension was heat-treated (5 min, 96°C), centrifuged (13,000 × g, 2 min), and a 5 μl portion of the supernatant was used as a source of genomic DNA for PCR amplification. The PCR primer sets used to amplify the coding region were designed to include at their 5’ a 15-nucleotide extension with sequence homology to the cloning site of the pET28b-Tev plasmid. The primers used for Preq were SY120: 5’-CAGGGCGCCATGTCCatgaaaaaaaatgcgagccttttaataac and SY121: 5’- CTCGAGTGCGGCCGCtcattgcatgcagatgtcactacacttcg; for Pdeg SY122: 5’- CAGGGCGCCATGTCCatgttaaataaaataattttaattactgg, and SY123: 5’- CTCGAGTGCGGCCGCtcatcgcaaaaaccctccttttaatag. Individual genes (Preq or Pdeg) were PCR-amplified in a 20 μl reaction volume that included buffer, dNTP’s (0.4 μl of 10 mM), Bacillus cereus genomic DNA (5 μl), PCR primer sets (1 μl each of 10 μM), and high fidelity Pyrococcus DNA polymerase (0.4U Phusion Hot Start II; New England Bioloabs). The PCR thermocycle conditions were 1X 98°C denaturation cycle for 30 sec followed by 25X cycles (each of 8 sec denaturation at 98°C; 25 sec annealing at 50°C; 30 sec elongation at 72°C), and finally 4°C. A similar PCR reaction was used to amplify the expression plasmid (pET28b-Tev) using a specific inverse-PCR primer set (SY118: GGACATGGCGCCCTGAAAATACAGGTTTTC and SY119: GCGGCCGCACTCGAGCACCACCACCACC) located near the NcoI and HindIII sites, respectively) with 25 sec annealing cycle at 58°C and 3 min elongation at 72°C. After PCR, a portion (4 μl each) of the amplified plasmid and insert were mixed, digested with 10U DpnI (15 min, 37°C), and then transformed into DH10B competent cells. Clones were selected on LB agar containing kanamycin (50 μg/ml) and positive clones were verified by PCR and by DNA sequencing using primers (T7 promoter and T7 terminator) flanking the gene insert. DNA sequences of the cloned genes were deposited in Genbank (with respective accession numbers KR012645 and KR012646). The recombinant DNA coding sequence was cloned to yield a recombinant protein fused at the N-terminal to a short peptide linker of 6 histidines (His_6_) followed by a TEV recognition amino acid sequence.

### Expression and Purification of Recombinant Pdeg and Preq

E. coli strains containing either pET28b-His_6_-TEV-Bc3750 (Pdeg), pET28b-His_6_-TEV-Bc3749 (Preq), or a non-related gene in pET plasmid that served as control, were cultured in separate flasks of 20 ml LB medium containing kanamycin (50 μg/ml) and chloramphenicol (35 μg/ml). The cells were grown for 20 hours while shaking at 250 rpm at 37°C. A 5 ml of each culture was transferred to a separate flask containing 250 ml LB medium supplemented with the same concentrations of antibiotics, and grown at 37°C while shaking at 250 rpm until the cell densities reached A_600_ = 0.6. The cells were then induced by the addition of isopropyl ß-D-1-thiogalactopyranoside (IPTG) to a final concentration of 0.5 mM and grown for 20 hours at 18°C while shaking at 225 rpm. Each culture was then centrifuged (6,000 x g for 10 min at 4°C), and cell pellet was washed with 20 ml cold double-distilled water and recentrifuged. The cell pellets derived from the IPTG-induced culture were resuspended in 10 ml cold lysis buffer [50 mM Tris-HCl pH 8.3, 30% glycerol, 50 mM KCl, 20 mM ß-mercaptoethanol (BME)]. The resuspended cells were kept on ice and sonicated (10 sec pulse and 20 sec off) using a Misonics S4000 Sonicator for 12 cycles at 30% amplitude. Following sonication, the lysed cell solution was centrifuged (6,000 x g for 15 min at 4°C), and the supernatant (labeled S6) was collected, supplemented 2 mM BME, and centrifuged (20,000 x g for 30 min at 4°C). The resulting supernatant (labeled S20) was collected, divided to smaller aliquots, flash frozen in liquid nitrogen, and then stored at -80°C until Ni-purification.

The His-tagged recombinant proteins (Pdeg, Preq, and the control unrelated 6His-protein) were purified over a Fast-Flow Ni^2+^-Sepharose column [2 ml of resin packed in a 1 cm inner diameter x 10 cm long column). Before purification, each column was equilibrated with Buffer A (50 mM Tris-HCl pH 8, 10% glycerol, 100 mM NaCl, 5 mM BME). The frozen S20 fraction of recombinant Pdeg (Bc3750), Preq (Bc3749), or control was rapidly thawed, and 7.5 ml was loaded onto the Ni-column. Unbound protein was washed from the column with 7.5 ml of Buffer A followed by 15 ml Buffer E1 (Buffer A containing 10 mM imidazole-HCl pH 8). Subsequently, the column was washed with 20 ml Buffer E4 (Buffer A containing 60 mM imidazole-HCl pH 8), and 7.5 ml Buffer E7 (Buffer A containing 250 mM imidazole-HCl pH 8, and 5 mM BME). The activity of recombinant Pdeg and Preq typically eluted with E7 buffer. After elution, these fractions were supplemented with an additional 0.75 ml 80% glycerol, and Pdeg was further supplemented with an additional 133 μM NADP^+^. Following supplement addition, the protein fractions were flash frozen in liquid nitrogen and stored at -80°C. The concentration of purified protein was determined at 280 nm using a theoretical extinction coefficient (http://web.expasy.org/protparam/) of ϵ = 23,630, and 30,285 m^−1^ cm^−1^, based on the amino acid sequence of Pdeg (Bc3750) and Preq (Bc3749), respectively. The molecular mass of purified protein was estimated after separation on SDS-PAGE and Coomassie blue staining. The potential size of the purified enzyme in native condition was estimated by chromatography on a gel filtration column (Superdex 75, 1x30 cm; GE Healthcare) and UV detection at 215 nm. The Superdex column was equilibrated with cold buffer [10 mM Tris-HCl (pH 7.0), 150 mm NaCl] at a flow rate of 0.5 ml/min. Size of protein was determined using known molecular weight of marker proteins as standards.

### Enzymatic Assays and Product Analysis of Pdeg and Preq

For Pdeg, the activity was examined by time-resolved ^1^H-NMR spectroscopy and by HILIC-HPLC with UV or electro spray ionization mass spectrometry (ESI-MS). For HPLC-based assays, the reaction assay, in a total volume of 50 μl, contained 50 mM Tris-HCl pH 8.0, 1 mM UDP-GlcNAc, 1 mM NADP^+^, and 400 pM of recombinant Pdeg. The reaction was terminated by heating for 2 min at 95°C. Chloroform was then added as described [18] and an aliquot (30 μl) of the upper layer phase was removed and mixed with 57 μl acetonitrile and 3 μl 0.5 M ammonium-acetate pH 4.35. A portion (10 μl) of this mixture was analyzed using HILIC chromatography coupled to an ESI-MS/MS. HPLC-ESI-MS/MS was carried out on the Shimadzu ESI-IT-TOF using a Nexera LC-30AD pump, autosampler (Sil30), and column heater (at 37°C). The column used for separation was an Accucore 150-amide HILIC column (150×4.6 mm, 2.6 μm particle size, ThermoScientific). The chromatography gradient conditions for the HPLC-ESI-MS were composed of two solvents: Solvent A, 40 mM ammonium-acetate, pH 4.35 (A) and solvent B, acetonitrile (ACN). The column was equilibrated at 0.7 ml/min with 25% A and 75% B. After injection, chromatography conditions were: from 0–2 min, flow rate 0.7 ml/min at 25% A/75% B, followed by a 23 min gradient to 65% A, then a 10 min gradient to 50% A. Subsequently, column was washed for 10 min with 25% A/75% B prior to the next injection. To detect analytes, the MS detector was operated in the negative mode with detector voltage 1.75 kV. In addition to ESI-MS analysis, another portion of each enzyme assay (10 μl, prepared as above) was subjected to analysis by an HPLC-UV system. This chromatographic system included an Agilent 1260 pump, autosampler, column heater (at 40°C), and a diode array detector (UV). For chromatographic separation, a narrow-bore Accucore 150-amide HILIC column (250x2.1 mm, 2.6 μm particle size) was used with a gradient elution method consisting of 40 mM ammonium acetate buffered to pH 4.35 (solvent A) and acetonitrile (solvent B). After equilibration at 0.25 ml/min with 25% A (75% B), the gradient elution method was programmed to run at 25% A and 0.25 ml/min from 0 to 10 min, increase (linearly) to 39% A from 10 to 30 min, increase to 50% A (and decrease flow rate to 0.2 ml/min) from 30 to 35 min, run at 50% A from 35 to 40 min, decrease to 25% A (and increase flow rate to 0.25 ml/min) from 40 to 45 min, and run at 25% A from 45 to 60 min. Peaks of enzymatic products detected by A_261_ (max for UDP-sugars) were collected for further analyses.

For the NMR-based assays of recombinant Pdeg, a reaction in a volume of 180 μl consisted of 120 μl of D_2_O and 60 μl of water-based reagents [50 mM Tris-HCl, pH 8.0, 1 mM UDP-GlcNAc, 400 pM of purified Pdeg, and 1 mM DSS (2,2-dimethyl-2-silapentane-5-sulfonate) that served as internal NMR standard]. The reaction mixture was transferred to a 3-mm NMR tube, and the products were analyzed by ^1^H-NMR spectroscopy for up to 3 hours at 25°C using a Varian DirectDrive 600-MHz spectrometer equipped with a cryogenic probe. For the NMR-based Preq assay, 105 pM of purified recombinant Preq was added along with 9 μl of 10 mM NADPH to a 180 μl Pdeg reaction. The products were analyzed for up to 1 hour at 25°C in the same 600-MHz spectrometer. Data were acquired before the addition of the enzyme as time zero (t0). After adding the enzyme, acquisition was started after ~3 min to allow the spectrometers operating parameters to be optimized. Sequential one-dimensional proton spectra with presaturation of the water resonance were acquired over the course of the reaction. All NMR spectra were referenced to the resonance of DSS set at 0.00 ppm. Processing of the data was performed with MestreNova (MestreLab Research).

### NMR Analysis of Enzymatic Products

Individual enzyme reaction products were collected based on their UV absorbance during chromatography with HILIC HPLC-UV. After lyophilization, the UDP-sugar products were dissolved in 100% D_2_O and characterized by NMR. 2D-NMR spectra were obtained at room temperature on Varian INOVA 600 and 900 MHz spectrometers. Each purified UDP-sugar was identified using COSY [19], TOCSY [20] (80 ms mixing time), HSQC [21], and HSQC-TOCSY [22] with 80 ms mixing time.

### Characterization and Kinetic Analyses of the Recombinant Enzymes Pdeg and Preq

Various assays were carried out to determine a suitable temperature, pH, and buffer for optimal activity of both recombinant enzymes. To establish an ideal temperature for optimal activity of Pdeg, a standard reaction assay was performed at various temperatures between 4°C and 42°C for 2 hours. The standard reaction assay contained 400 pM of purified recombinant Pdeg, 1 mM UDP-GlcNAc, 0.5 mM NADP^+^, and 50 mM Tris-HCl pH 8. The amount of product formed over time was determined by HPLC-UV. The optimal temperature for recombinant Preq was determined by performing a standard reaction assay at various temperatures. To obtain UDP-4-keto-6-deoxy-D-GlcNAc substrate for Preq kinetic reactions, a larger scale Pdeg reaction was first carried out. This reaction consisted of 50 mM Tris-HCl pH 8, 4 mM UDP-GlcNAc, 1 mM NADP^+^, and 1.6 nM purified recombinant Pdeg. The reaction was incubated overnight for maximum conversion of the product to substrate and the concentration of the UDP-4-keto-6-deoxy-D-GlcNAc product was determined after elution from a HILIC column with UV detection.

The activities of Pdeg and Preq in different buffers (Tris-HCl and sodium phosphate) at pH values between 6 and 10 were also investigated. Assays were performed at 22°C for 15 min (Pdeg) or 75 sec (Preq). The assays specific to Pdeg contained 400 pM of recombinant enzyme, 0.5 mM UDP-GlcNAc, 0.2 mM NADP^+^, and 50 mM of a given pH-adjusted buffer. Assays specific to Preq contained 105 pM of recombinant enzyme, 565 μM UDP-4-keto-6-deoxy-GlcNAc, 1 mM NADPH, and 50 mM of a given buffer. The amount of product formed (as detected via HPLC-UV) was used to determine optimal assay conditions.

Inhibition assays were performed by first mixing a potential inhibitor compound, recombinant enzyme, and buffer. After incubating on ice for 5 min, reaction substrates and additives were added to the reaction vial. The inhibition assays for Pdeg consisted of 50 mM Tris-HCl pH 8, 0.2 mM inhibitor, 0.5 mM UDP-GlcNAc, and 400 pM recombinant enzyme. The inhibition assays for Preq consisted of 50 mM Tris-HCl pH 8, 0.2 mM inhibitor, 565 μM UDP-4-keto-6-deoxy-GlcNAc, 1 mM NADPH, and 105 pM recombinant enzyme. Assays were performed at 22°C for 15 min (Pdeg) or 75 sec (Preq), and product formation was determined with HPLC.

Initial kinetic assays were performed in triplicates with different substrate concentration and the final kinetic assays for Pdeg were performed at 22°C for 15 min, since, between 10 and 20 min, product formation is linear across time. The final Preq kinetic studies were performed at 22°C, and the reaction was terminated after 105 sec. Between 60 and 120 sec, product formation of Preq is linear across time. The final kinetic studies to determine the k_cat_ and K_m_ of Pdeg, reaction assays consisted of various concentrations of UDP-GlcNAc (10, 20, 40, 80, 100, 160, 200, 300, 400, 500, 600, 700, 800, 900, and 1000 μM) with a fixed amount of co-factor (200 μM NADP^+^ and NADPH) and 400 pM of recombinant Pdeg. For Preq assays, the reaction included various concentrations of UDP-4-keto-6-deoxy-GlcNAc (23, 68, 113, 203, 248, 293, 339, 384, 429, 474, 519, 564, 609, 655, 700, 745, 790, 835, 880, and 925 μM) with a fixed amount of co-factor (2 mM NADPH) and 100 pM of recombinant Preq. The products formed were analyzed by HPLC and used to determine initial velocities. The kinetic parameters were derived by fitting enzyme kinetic curves with GraphPad Prism 5.

For Pdeg substrate specificity studies, assays in a final volume of 50 μl consisted of 50 mM Tris-HCl pH 8, 0.5 mM NADP^+^, 0.5 mM NADPH, 2 mM of various UDP-sugars (UDP-galactose, UDP-GalNAc, UDP-GlcNAc, and UDP-glucose), and 400 pM recombinant Pdeg and were performed for 4 hours at 22°C. For each reaction, amounts of product and substrate remaining were determined after chromatography via HILIC-HPLC detection.

## Results

### Identification of QuiNAc in Bacillus

In our systematic survey of prokaryotic glycans isolated from various Bacillus species, we unexpectedly identified an alditol-acetate 6-deoxy-2-N-acetylhexosamine sugar residue-derivative eluted from a GC-column at 29.7 min (S1 Fig). The electron ionization mass spectrometry (EI-MS) of this peak showed prominent fragment ions at m/z 302, 260, 201, 145, 129, 103, and 85 (see insert in S1 Fig) identical with those found for alditol acetates derivatives of a QuiNAc std. To the best of our knowledge, little was known about QuiNAc formation in gram-positive bacteria, and it inspired us to further investigate the metabolic pathway involved in biosynthesis of QuiNAc in Bacillus.

### Identification of Pdeg and Preq from Bacillus cereus ATCC 14579

To identify potential genes encoding enzymes involved in the formation of activated-QuiNAc, we first performed a BLAST search using amino acid sequences of known bacterial UDP-GlcNAc 4,6-dehydratases. This led us to identify B. cereus ATCC 14579 Bc3750 (herein referred to as Pdeg) as a candidate. Interestingly, the Bacillus Pdeg protein shares a high amino-acid sequence homology with functional proteins determined to have not only a C4,6-dehydratase but also a C5-epimerae activity. As shown in Table 1A, Pdeg protein has a high amino acid sequence identity (46%) with functional UDP-GlcNAc-5-inverting-C4,6-dehydratase, FlaA1 (PseB, in Campylobacter jejuni), from Helicobacter pylori [23], a 41% amino acid sequence identity with CapE of Staphylococcus aureus [24] and 35% similarity to Mg434 from the giant virus Megavirus chilensis [25]. It is worth noting that Pdeg has much lower amino acid sequence identities, 27%, 34%, 37%, and 34% with several functional UDP-N-acetyl-glucosamine-C4,6-dehydratase (lacking 5-epimerase) from Vibrio Fischeri [26], with PglF, involved in UDP-diNAcBac biosynthesis pathways from Campylobacter jejuni [27], with PglD from Neisseria gonorrhoeae [28], with WEEK from Acinetobacter baumannii [29], respectively, as shown in Table 1A. Here, we provide biochemical evidence showing that despite higher sequence similarity to a dual 5-epimerase, C4,6-dehydratase enzymes, Bacillus protein Pdeg is a 4,6-dehydratase with no detectable 5-epimerase activity. This suggests that protein sequence homology is not sufficient to determine activity of an unknown gene. Flanking Pdeg (Bc3750) in Bacillus cereus ATCC 14579 is gene Bc3749 (herein referred to as Preq, Fig 1B). Preq and its adjacent gene unit Pdeg are overlapped by 4 nucleotides. Such gene overlap and reading frame offset appeared conserved in other Bacilli including, for example, Bacillus weihenstephanensis FSL R5-860, Bacillus cereus ATCC 10876, Bacillus thuringiensis serovar israelensis ATCC 35646, and Bacillus thuringiensis serovar kurstaki str. HD-1. It is therefore possible that the expression of the Pdeg and Preq gene unit is under the same regulation mechanism. The Preq protein homology to other known enzymes is not clear. As shown in Table 1B, Preq shares, for example, 33% amino acid sequence identity with functional GDP-4-keto-6-deoxy-D-mannose 4-reductase from Aneurinibacillus thermoaerophilus [30] and lower sequence homology (24%) with dTDP-glucose 4,6-dehydratase (Rmlb) from Salmonella Enterica serovar Typhimurium [31]. Also, it shares lower sequence homology (29%) with functional UDP-2-acetamido-2,6-dideoxy-D-xylo-4-hexulose-4-reductase from Rhizobium etili [32] even though its function, as we will described, is the same with Bacillus protein Preq. Below we provide biochemical evidences of Pdeg (Bc3750) and Preq (Bc3749) proteins for their sequential ability to convert UDP-GlcNAc to UDP-4-keto-6-deoxy-GlcNAc and to UDP-QuiNAc.

**Table 1:** Comparative analysis of *Bacillus cereus* ATCC 14579 proteins Pdeg (Bc3750) and Preq (Bc3749) with known genes encoding UDP-sugar 4,6-dehydratase (A) and 4-reductase (B) from bacterial sp.

**A**.	Accession (name)	E-Value	AA Identity	Function in Species
Pdeg	YP_203523	3e^-18^	27%	UDP-N-acetyl-glucosamine 4,6-dehydratase (Vibrio Fischeri)
Pdeg	CJ1293 (PseB)	4e-77	47%	UDP-GlcNAc-specific C4,6 dehydratase/C5 epimerase (Campylobacter jejuni)
Pdeg	HP0840	5e-83	46%	UDP-GlcNAc-inverting 4,6-dehydratase (Helicobacter pylori)
Pdeg	3VVB_A (CapE)	8e^-75^	41%	UDP-GlcNAc 5-inverting 4,6-dehydratase (Staphylococcus aureus)
Pdeg	4TQG (Mg434)	6e^-55^	35%	UDP-GlcNAc 5-inverting 4,6-dehydratase (Megavirus chilensis)
Pdeg	YP_471773.1	8e^-48^	34%	UDP-N-acetyl-glucosamine 4,6-dehydratase (Rhizobium etli)
Pdeg	CAL35237.1	5e^-45^	34%	PglF (Campylobacter jejuni)
Pdeg	YP_207256.1	8e^-48^	37%	PglD (Neisseria gonorrhoeae)
Pdeg	AHB32380.1	1e^-48^	34%	WeeK (Acinetobacter baumannii)
**B**.	Accession	E-Value	AA Identity	Function in Species
Preq	Q6T1X6	5e^-44^	33%	GDP-4-keto-6-deoxy-D-mannose 4-reductase (Aneurinibacillus thermoaerophilus)
Preq	1BXK	2e^-14^	24%	dTDP-glucose 4,6-dehydratase (Salmonella Enterica serovar Typhimurium)
Preq	YP_470339.1	2.4	29%	UDP-2-acetamido-2,6-dideoxy- D-xylo-4-hexulose 4-reductase (Rhizobium etli)
Preq	AAF23991	2.9	28%	4-reductase (P. aeruginosa O6)

### Bc3750 (Pdeg) encodes a UDP-GlcNAc-C4,6-Dehydratase

**Fig 2:**
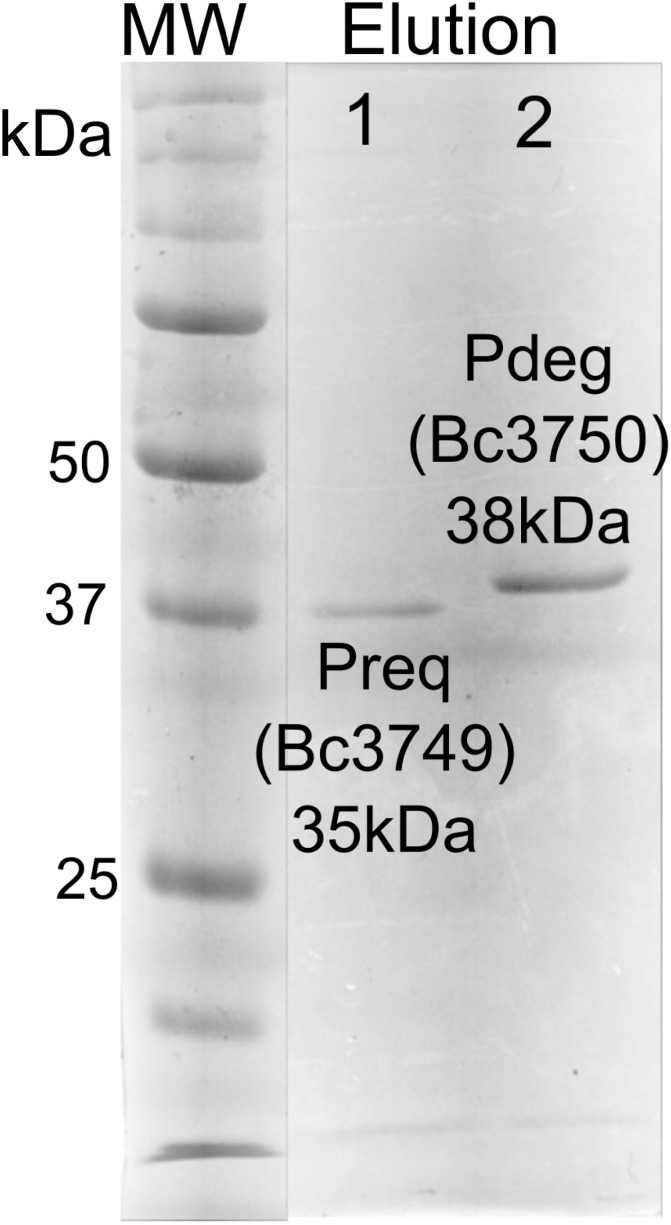
SDS—PAGE analysis of the B. cereus ATCC 14579 purified recombinant Bc3750 (Pdeg) and Bc3749 (Preq) proteins involved in the biosynthesis of UDP-QuiNAc. Protein standards are shown on the right in kDa. The final elution fraction (E7) of purified recombinant proteins from affinity column is shown for Bc3749 (lane 1) and Bc3750 (lane 2).

**Fig 3:**
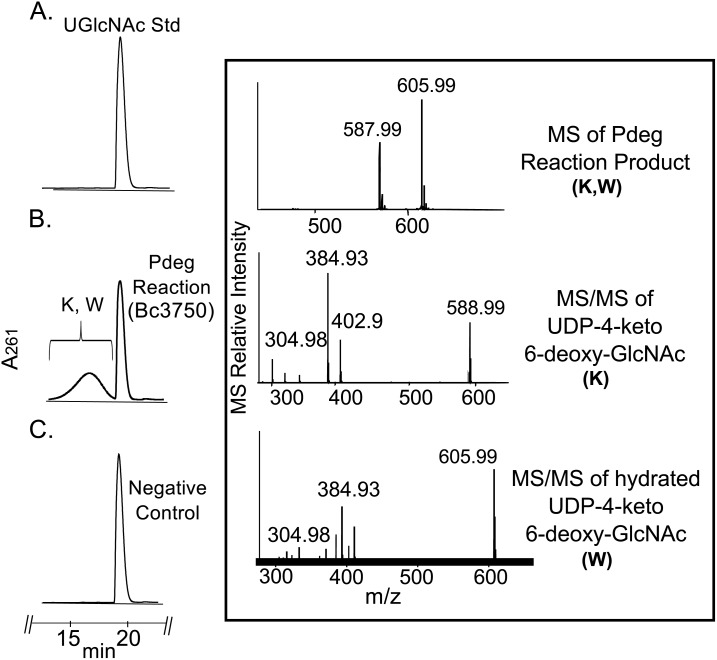
Analysis of recombinant enzyme reaction Pdeg by UV-HPLC and LC-ESI-MS-MS. A. UDP-GlcNAc standard reaction is shown. B. UDP-GlcNAc C4,6-dehydratase reaction, is the conversion of UDP-GlcNAc to UDP-4-keto-6-deoxy-HexNAc. The broad peak (K and W) denotes 4-keto and 4-hydrated-keto form of the UDP-4-keto-6-deoxy-sugar. Boxed top panel shows the product ions, K and W, m/z 587.99 and 605.99, respectively. MS-MS analysis of parent ions K and W gave fragment ions at m/z 402.9, 384.9 and 304.9 that are consistent with [UDP-H]^-^, [UDP-H_2_O-H]^-^, and [UMP-H_2_O-H]^-^, respectively. (Boxed the second and third panel) C. Pdeg negative control reaction carried out with unrelated protein.

**Fig 4:**
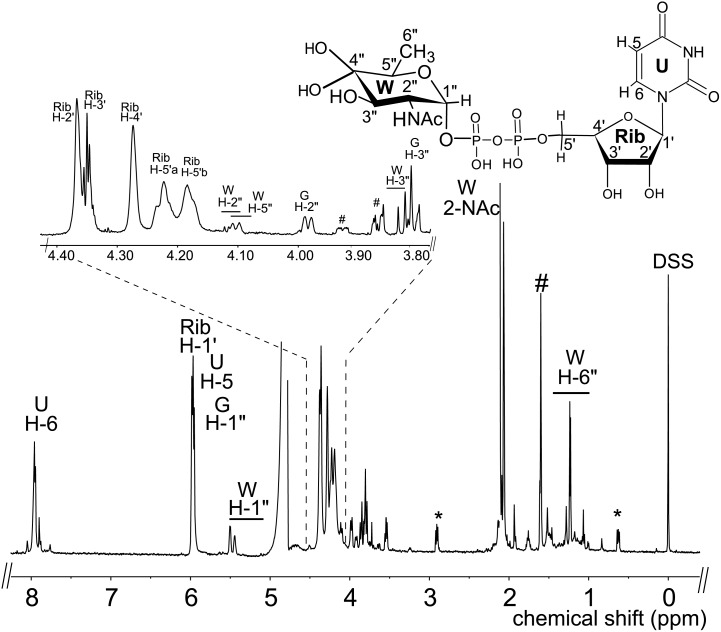
Analysis of Pdeg recombinant enzyme reaction products by ^1^H-NMR indicates formation of hydrated-UDP-4-keto-6-deoxyl-D-GlcNAc. The product peak of the Pdeg reaction was collected and analyzed at 600 MHz NMR. Full proton spectrum of the Pdeg product hydrated-UDP-4-keto-6-deoxy-D-GlcNAc. Expanded proton spectra between 3.8 and 4.4 ppm that shows the sugar ring. The short line above NMR ‘peaks’ denotes specific chemical shifts belonging to a UDP-4-keto-6-deoxy-D-GlcNAc. Symbol(#) denotes column contamination and symbol (*) denotes DSS.

E. coli cells expressing recombinant His6-Bc3750 (Pdeg) were used to isolate and purify the recombinant protein using a Ni-affinity column (Fig 2, lane 2, calculated 38 kDa based on amino-acid sequence). Initial enzyme characterization of purified Pdeg was determined by a UV-HPLC and ESI-mass spectrometry. HILIC analysis of the enzymatic products formed when purified Pdeg was reacted with UDP-GlcNAc showed the appearance of a new broad peak with a retention time of 16 min (Fig 3 panel B, labeled K and W). This peak was not detected in a reaction with unrelated protein (Fig 3 panel C). When the Pdeg enzymatic reactions were chromatographed and analyzed in the negative mode by ESI-MS, the broad peak (K, W) gave two major ions with m/z 587.99 and 605.99 (Fig 3 Box top panel). These m/z values likely correspond to [M-H]^-^ for a UDP-4-keto-6-deoxy-HexNAc and the hydrated form of the UDP-4-keto-6-deoxy-HexNAc. MS-MS analysis of K parent ion gave fragment ions at m/z 402.9, 384.93 and 304.98 that are consistent with [UDP-H]^-^, [UDP-H_2_O-H]^-^, and [UMP-H_2_O-H]^-^, respectively. The neutral loss of 185 mass unit from m/z 588 implies a mass for a 4-keto-6-deoxy-HexNAc sugar. These initial analyses led us to suspect that the newly formed Pdeg enzymatic product is a UDP-4-keto-6-deoxy-HexNAc. However, the sugar configuration could not be determined by MS analyses, so the product peak was collected by HPLC and analyzed by NMR spectroscopy. Chemical shift assignments obtained by one-dimensional and two-dimensional NMR experiments and coupling constants (Table 2 and Fig 4) indicated that the enzymatic product is UDP-4-keto-D-GlcNAc. The 4-keto-6-deoxy-GlcNAc H1” anomeric region of the proton spectrum contains a quadruplet signal with chemical shifts of 5.44 ppm. The distinct chemical shift of the anomeric proton and the coupling constant values of 3 Hz for J_H1”, H2_” and 7.1 Hz for J_H1”,P_ are consistent with an α-linkage to the phosphate of UDP. The chemical shifts for each H6” has a value of 1.23 ppm. The methyl protons resonance of the N-acetylated group (C-2”-NAc) at 2.07 ppm is consistent for a C2 acetamido moiety of the product. The D-configuration of the linked sugar was established based on coupling constants. An L-sugar would have larger coupling between α-phosphate and H1” proton and also larger coupling between H2” and H3” [33]. Further support for the presence of the UDP-4-keto-6-deoxy-D-GlcNAc product was established using two-dimensional NMR experiments. COSY and TOCSY experiments confirmed the assignments for H2”, H3”, and H5” in this 4-keto-6-deoxy sugar. The J_H2”, H3”_ coupling constant of 10 Hz and the J_H3”, H5”_ coupling constant of 10 Hz is consistent with a gluco-configuration. ^13^C-HSQC and HSQC-TOCSY experiments established carbon assignments and the presence of an N-acetylated carbon.

**Table 2:** NMR data for the sugar moiety of UDP-4-keto-6-deoxy-GlcNAc (W) and UDP-QuiNAc (Q).

Hydrated-UDP-4-keto-6-deoxy-GlcNAc (W)			UDP-QuiNAc (Q)		
^1^H	Chemical Shift (ppm)	Coupling Constant (Hz)	^1^H	Chemical Shift (ppm)	Coupling Constant (Hz)
H1”	5.44	J_1,p_ (7.1)	H1”	5.44	J_1,p_ (7.18)
H2”	4.11	J_1,2_ (3.5)	H2”	3.99	J_1,2_ (3.6)
H3”	3.81	J_2,3_ (10)	H3”	3.74	J_2,3_ (10)
H4”			H4”	3.25	J_3,4_ (10)
H5”	4.11		H5”	3.99	J_4,5_ (9.8)
H6”	1.23	J_5,6_ (6.2)	H6”	1.29	J_5,6_ (6.0)
NAc-H”		2.07	NAc-H”	2.06	

**Fig 5:**
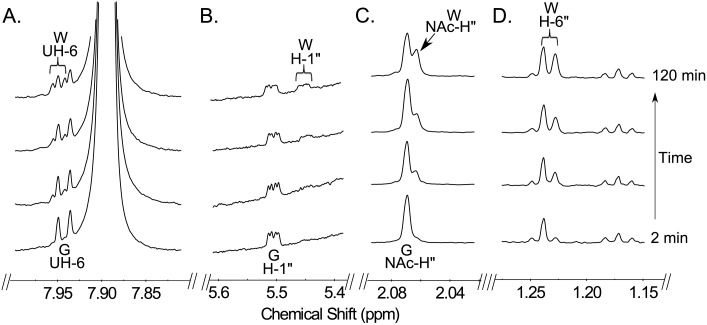
Time-resolved ^1^H-NMR analysis of Pdeg reaction showing the conversion of UDP-GlcNAc to hydrated-4-keto-UDP-suagr. Spectra were collected for the first 120 min of the reaction that was conducted at 25°C and included Pdeg and 1 mM of UDP-GlcNAc (time 0). Four selected regions of the UDP-4-keto-6-deoxy-GlcNAc formed over time, can be observed with a diagnostic UH-6 (A), anomeric proton (B), acetyl methyl proton (C), and 6-deoxy proton H6’ (D). Proton signals of UDP-GlcNAc and product, UDP-4-keto-6-deoxy-HexNAc, are labeled as GUH-6, GH-1”, GNAc-H” for the substrate UDP-GlcNAc, WUH-6, WH-1”, WNAc-H” and WH-6” for the hydrated form, respectively.

We next used time-resolved ^1^H NMR to monitor the conversion of UDP-GlcNAc to UDP-4-keto-6-deoxy-GlcNAc with purified recombinant Pdeg (Fig 5). The C4,6-dehydratase reaction generates the C4”-hydrated form of UDP-4-keto-6-deoxy-GlcNAc (W in Fig 5). When the recombinant UDP-GlcNAc 4,6-dehydratase is added, additional signals corresponding to the anomeric proton of C4”-hydrated form of UDP-4-keto-6-deoxy GlcNAc (WH-1”) appear and are accompanied by a decrease in the intensity of the signals corresponding to the anomeric protons of GlcNAc (GH-1”) (Fig 5B). Also, the 6-deoxy proton signal (WH-6”) starts showing up at 1.23 ppm, while other signals close to the 1.17 ppm do not change (See Fig 5D). Time-resolved NMR also detected a chemical shift change of protons of uracil and the NAc methyl group (Fig 5A and 5C). Together, these data confirm that Bc3750 (Pdeg) encodes a UDP-GlcNAc-C4,6-dehydratase that converts UDP-D-GlcNAc to UDP-4-keto-6-deoxy-GlcNAc (Fig 1A).

### Bc3749 (Preq) Encodes C4”-reductase and forms UDP-QuiNAc

**Fig 6:**
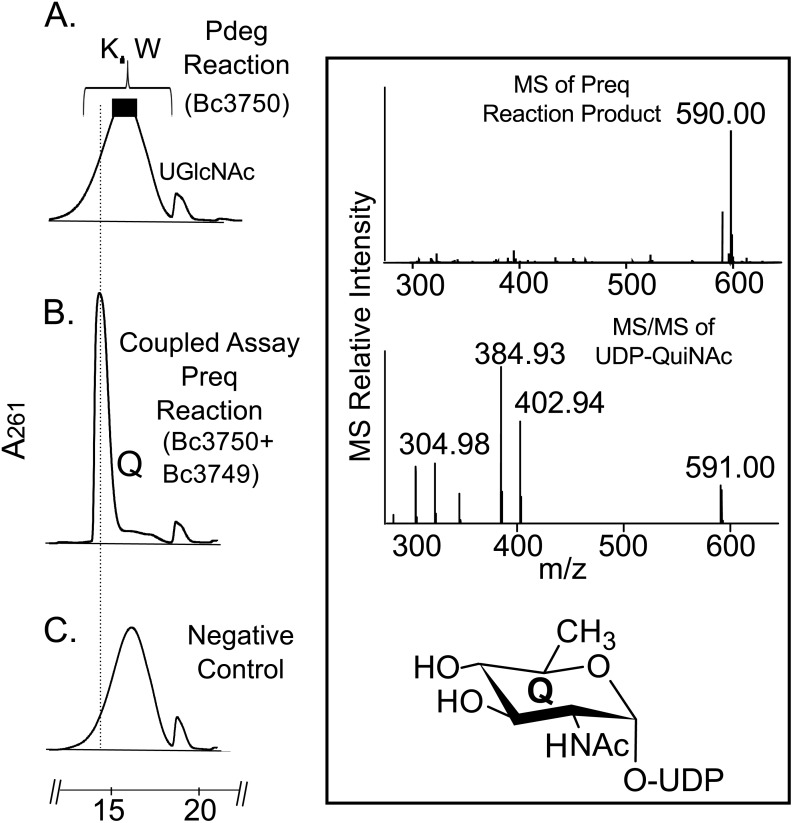
Analysis of Preq recombinant enzyme reaction by UV-HPLC and LC-ESI-MS-MS. A. Pdeg reaction shows the conversion of UDP-GlcNAc to UDP-4-keto-6-deoxy-HexNAc before recombinant Preq addition. B. Following Pdeg reaction, purified Preq was added and reacted with UDP-4-keto-6-deoxy-GlcNAc in the presence of NADPH to give product Q, UDP-QuiNAc, with m/z 590 and ms/ms of 402.94, 384.93, and 304.98 (Boxed top and second panel) C. Preq negative control reaction carried out with unrelated protein.

**Fig 7:**
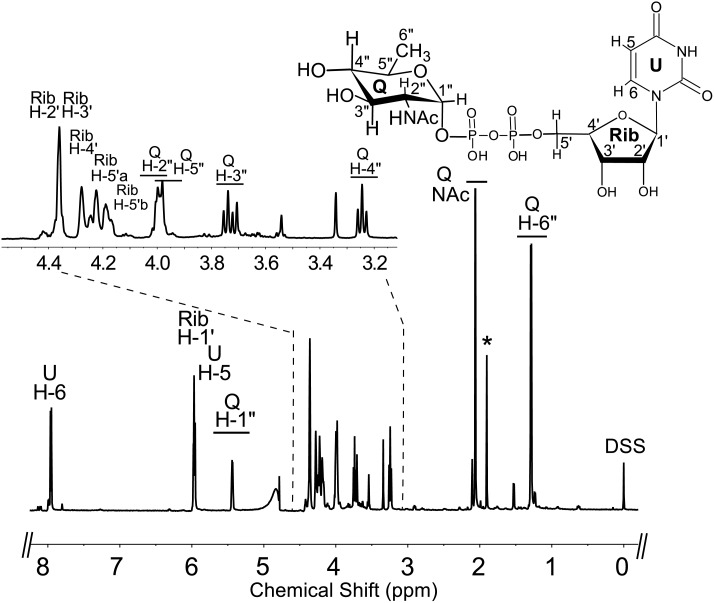
Analysis of Preq reaction product by ^1^H-NMR indicates formation of UDP-QuiNAc. The product of the Preq reaction (peak Q, in Fig 6 Panel B) was collected and analyzed at 600 MHz NMR. Full proton spectrum of HPLC-collected product UDP-QuiNAc. Expanded proton spectra between 3.2 and 4.4 ppm that shows the QuiNAc sugar ring. The short line above NMR ‘peaks’ denotes specific chemical shifts belonging to a UDP-QuiNAc.

**Fig 8:**
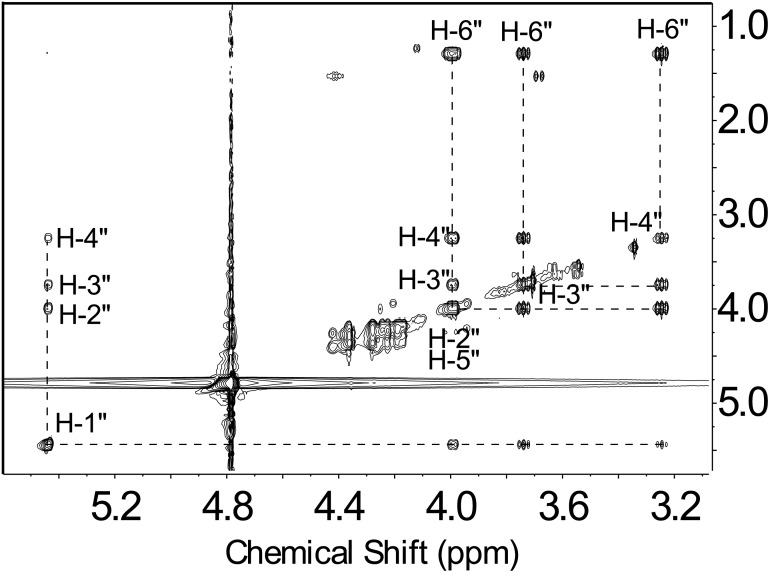
TOCSY spectrum of UDP-QuiNAc shows the coupling among QuiNAc sugar ring protons.

To determine the function of Preq, E. coli cells expressing the gene were used to isolate and purify the recombinant protein (Fig 2, lane 1; calculated 35 kDa). During the initial characterization of the Preq protein, we determined that activity required NADPH and UDP-4-keto-6-deoxy-sugar, as no activity was observed without NADPH or with non-keto-sugars. As shown in Fig 6 (panel B, labeled Q) purified recombinant Preq in the presence of NADPH readily converted UDP-4-keto-6-deoxy-GlcNAc to a new UV-peak migrating on a HILIC column with a retention time of 14.9 min (Fig 6. panel B). This peak was not formed with unrelated protein (negative control, Fig 6. panel C). Negative ion LC-ESI-MS analyses of the new product gave a major ion at m/z 590 (Fig 6. Box top panel), suggesting the keto moiety is reduced. MS-MS analysis of m/z 591 gave ion fragments at m/z 402.94, 384.93 and 304.98 consistent with [UDP]^-^, [UDP-H_2_O]^-^, and [UMP-H_2_O]^-^ respectively. The neutral loss of a 187 mass unit implies a mass for a 6-deoxy-HexNac sugar. One-dimensional (Fig 7) and two-dimensional NMR (Fig 8) spectroscopic analyses of the isolated product provided compelling evidence that recombinant enzyme, Preq, formed UDP-D-QuiNAc. The chemical shift assignments for UDP-QuiNAc, are summarized in Table 2. NMR signals at 56.57 ppm for C_2_ of the sugar moiety established that it is substituted by nitrogen atoms. A ^1^H signal at 2.06 ppm also indicated that the C_2_ position of the sugar was N-acetylated. The J_H2”, H3”_ and J_H3”, H4”_ coupling constants of 10 Hz and 10 Hz, respectively, are consistent with a gluco sugar. The chemical shift of 5.44 ppm of the anomeric proton of the QuiNAc residue and J_H1”,P_ and J_H1”, H2”_ coupling constants of 7.18 and 3.0 Hz, respectively, are consistent with an α-linkage. Collectively, we concluded that Preq encodes an enzyme that reduces UDP-4-keto-6-deoxy GlcNAc to form UDP-QuiNAc.

**Fig 9:**
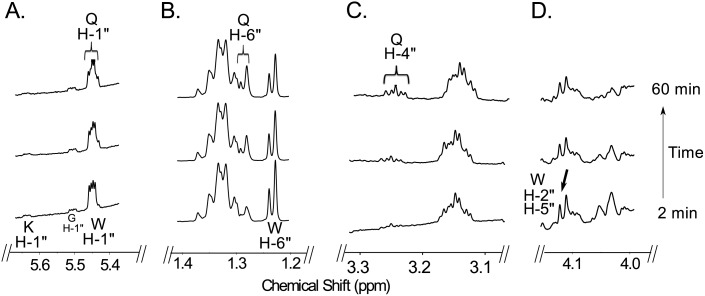
Time-resolved ^1^H-NMR analysis of Preq reaction showing the conversion of hydrated-UDP-4-keto-6-deoxy-GlcNAc to UDP-QuiNAc. Spectra were collected for the first 60 min of the reaction that was conducted at 25°C and included Preq and 1 mM NADPH after Pdeg reaction. Four selected regions of the UDP-QuiNAc formed over time, can be observed with a diagnostic anomeric proton QH-1” (A), 6-deoxy proton QH-6” (B), appearance of QH-4”. Proton signals of UDP-QuiNAc are labeled as QH-1”, QH-6”, and QH-4” and disappearance of WH-2” and WH-5” (D). Note that the chemical shift of the anomeric proton of UDP-QuiNAc (QH-1”) is very close to the proton of UDP-4-keto-6-deoxy-GlcNAc (slightly shifted). WH-1” is the hydrated form of UDP-4-keto-6-deoxy-GlcNAC.

Time-resolved ^1^H NMR used to monitor the reaction progress of Preq is shown in Fig 9. As time progresses, the peak corresponding to the anomeric proton (WH-1”, see Fig 9A) of the UDP-4-keto-sugar substrate is decreased, whereas the peak of the enzymatic product, UDP-QuiNAc (QH-1”), is increased. It is also important to note that the UDP-4-keto intermediate (the product of recombinant Pdeg) is also disappearing during the formation of UDP-QuiNAc. The distinct chemical shifts for the proton signals belonging to the methyl group of the substrate and product are also an indication of the enzymatic reaction’s progress with the 6-deoxy (WH-6”) substrate peak decreasing at 1.23 ppm and the product methyl peak (QH-6”) of the QuiNAc increasing at 1.29 ppm (Fig 9B). Diagnostic peak QH-4” of UDP-QuiNAc also appears around 3.25 ppm (Fig 9C) while WH-2” and WH-5” of 4-keto-6-deoxy GlcNAc disappears (Fig 9D).

### Enzymatic Properties of Pdeg and Preq

The recombinant Pdeg 4,6-dehydratase had its highest activity between 22 and 25°C and at pH 8 and 9 irrespective of the buffer used. Similar pH and temperature profiles were observed for Preq. Kinetics parameters for the recombinant Pdeg and Preq activities are summarized in Table 3. Recombinant Pdeg eluted from a Superdex 75 size-exclusion column in the region for a protein with a mass of ~26.7 kDa, suggesting that the enzyme is active as a monomer. Similarly, recombinant Preq eluted from the same column in the region for a protein with mass of ~26.8 kDa, implying this enzyme is active predominantly as a monomer. Further kinetics analyses of the recombinant Pdeg and Preq activities are summarized in Table 3. The apparent K_m_ values were 486.1 μM and 1,544 μM for UDP-sugar substrates, the V_max_ values were 7.4 μM min^-1^ and 2.3 μM min^-1^, and the k_cat_/K_m_ values were 38.3 μM^-1^min^-1^ and 15.1 μM^-1^min^-1^ with Pdeg and Preq, respectively. None of the UDP-sugars tested including UDP-galactose, UDP-GalNAc, UDP-GlcNAc, and UDP-glucose are substrates for recombinant Pdeg. Inhibition studies showed that UDP-galactose, UDP, UDP-glucose, UDP-GalNAc, and UTP reduced recombinant Pdeg activity by 51%, 77%, 81%, 91%, 98% respectively.

**Table 3:** Enzymatic properties of recombinant Bc3750-His_6_ (Pdeg) and Bc3749-His_6_ (Preq).

	Pdeg	Preq
Optimal pH^a^	8.0–9.0	8.0–9.0
Optimal temperature (°C)^a^	22–25	22
K_m_ (μM)^b^	486.1^c^	1544 ^c^
Vmax (μM-min^-1^)	7.4^c^	2.3^c^
kcat (min^-1^)	1.86x10^4^	2.34x10^4^
kcat/Km(μM^-1^ min^-1^)	38.3^c^	15.1^c^
Protein Mass (kDa)	37.2	35.0

^a^ Optimal pH and temperature assays were determined using Tris-HCl; the activity in pH 9 and 8 vary by less than 10%.

^b^ The reaction was determined by HPLC-UV after 15 min at 22°C incubation for Pdeg and 105 s at 22°C incubation for Preq. For Pdeg assays, the reaction consisted of various concentration of UDP-GlcNAc (10, 20, 40, 80, 100, 160, 200, 300, 400, 500, 600, 700, 800, 900, and 1000 μM) with fixed amount of co-factor (200 μM NADPH) and 0.75 ng recombinant Pdeg. For Preq assays, the reaction included various concentrations of UDP-4-keto-6-deoxy-GlcNAc (79, 237, 395, 553, 711, 869, 1027, 1185, 1343, 1501, 1659, 1817, 1975, 2133, 2291, 2449, 2607, 2765, 2923, 3081, and 3239 μM) with fixed amount of co-factor (2 mM NADPH) and 4 pg recombinant Preq.

^c^ The K and V values were derived by fitting enzyme kinetic curves with GraphPad Prism 5. Data were fitted with the best curve and sum of square calculated as 3.31 (for Pdeg) and 0.065 (Preq) and the relative standard deviation (RSDR) of Pdeg reaction and Preq reaction was calculated as 0.40 and 0.05, respectively.

## Discussion

In this study, we have identified two genes Preq and Pdeg (Bc3749 and Bc3750) in B. cereus ATCC 14579 that encode the enzymes capable of converting UDP-GlcNAc to UDP-QuiNAc (Fig 1). The first step is initiated by Bc3570 a specific C4,6-dehydratase, Pdeg, that generates the 4-keto sugar-nucleotide intermediate, UDP-4-keto-6-deoxy-GlcNAc. This intermediate exists in two forms: hydrated and keto forms. Existing in two forms appears to be a common feature observed with other 4-keto nucleotide-sugar derivatives including UDP-4-keto-6-deoxy-glucose [34], UDP-4-keto-6-deoxy-AltNAc [35], and UDP-4-keto-xylose [36]. At steady state the ratio of 4-hydrated to 4-keto is ~ 9:1. Hence, to distinguish the hydrated from the 4-keto-sugar-nucleotide by a typical proton NMR experiment is not obvious since no protons are detected at C-4 (keto or hydrated). Thus, one may report the chemical shifts for the hydrated rather the keto-form. An easy way to detect these two chemical forms is to carry the enzyme reaction in a time-resolved fashion or to analyze the enzyme reaction with a mass spectrometer (see Fig 3). Following the formation of the 4-keto nucleotide sugar, the second step catalyzed by Preq (Bc3749), reduces the C4-keto sugar moiety and converts it in an NADPH-dependent reaction to UDP-QuiNAc (Fig 9). The analog NADH could not be substituted for NADPH.

Despite the high amino acid sequence identity (46%) between the 4,6-dehydratases, Pdeg and PseB (C. jejuni), our experimental data shows that Pdeg does not have a 5-inverting (5’-epimerase) activity. While the amino acid sequence of the 4-reductase, Preq, indicated some similarities with proteins annotated as an epimerase, our biochemical analyses of the Preq enzymatic product data showed a 4-reductase activity forming only UDP-QuiNAc. The 4-epimer UDP-D-FucNAc was not produced by Preq under the experimental setup. When considering the data together we propose that the combined activities of Pdeg and Preq (as shown in Fig 1) contributes to synthesis of the UDP-QuiNAc in B. cereus and very likely in other Bacillus sp like Bacillus weihenstephanensis FSL R5-860, Bacillus cereus ATCC 10876, Bacillus thuringiensis serovar israelensis ATCC 35646, and Bacillus thuringiensis serovar kurstaki str. HD-1strains.

While this work was in progress, a study in the gram-negative, Rhizobium etili, discovered for the first time that this bacterium has two enzyme activities [32] identical to those as described here for the gram-positive bacterium, Bacillus. Both the rhizobium and Bacillus proteins have the same 4,6-dehydratase and 4-reductase activities to make UDP-QuiNAc. Interestingly, the 4-reductases from Rhizobium etli (YP_470339) and Pseudomonas aeruginosa O6 (WbpV, AAF23991.1) [37], share no sequence homology with the Bacillus enzyme. Furthermore, several UDP-GlcNAc-C4,6-dehydratases lacking 5’-epimerase activity from C. jeuuni, N. gonorrhoeae, and A. baumannii share much lower sequence homology with Pdeg enzyme (Table 1A). Current work is underway to determine the crystal structures of these Bacillus enzymes.

This illustrates that biochemical analysis is essential to assign function, and protein sequence homology is an insufficient criterion to infer functional specificity. The fact that Rhizobium 4-reductase requires NADH for its activity while Preq requires NADPH, could not explain in part the differences in primary sequences. Therefore, it is more likely that the genes encoding these activities in gram-positive and gram-negative bacteria are evolutionarily not related and perhaps arose from different ancestral genes.

## Supporting Information

S1 Fig Glycosyl residue compositions of QuiNAc-containing glycans in Bacillus sp. GC spectrum of alditol-acetates derived 6-deoxy-2-N-acetylhexosamine sugar residue eluted from GC-column at 29.7 min. The left box insert shows the electron impact mass spectrum and fragmentation (EI-MS) of this peak including prominent fragment ions at m/z 302, 260, 201, 145, 129, 103, and 85 identical with those found for alditol acetates derivatives of QuiNAc std. The right box insert shows the predicted primary and secondary MS fragments of C1-deuterated alditol-acetate of derived QuiNAc.
(TIFF)

S2 Fig TOCSY spectrum of UDP-4-keto-6-deoxy-GlcNAc shows the coupling among keto sugar ring protons. (TIFF)
