# Supplementary figures and images for: The Biosynthesis of UDP-d-QuiNAc in Bacillus cereus ATCC 14579
Source: PLoS One. 2015 Jul 24;10(7):e0133790. doi: 10.1371/journal.pone.0133790 (PMC4514872; doi:10.1371/journal.pone.0133790)

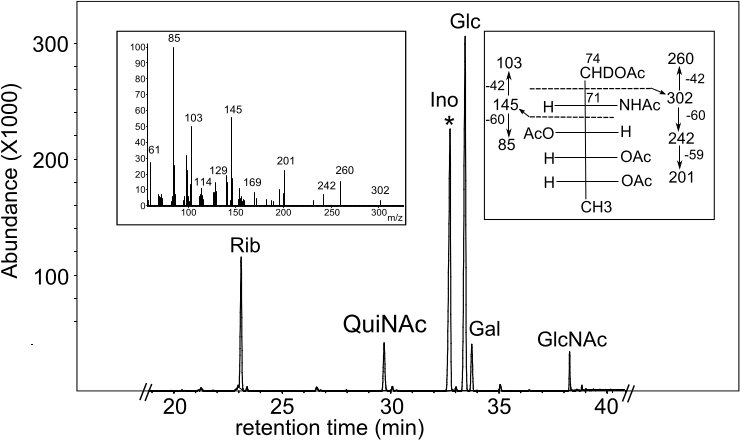

Supplement: S1 Fig — GC spectrum of alditol-acetates derived 6-deoxy-2-N-acetylhexosamine sugar residue eluted from GC-column at 29.7 min. The left box insert shows the electron impact mass spectrum and fragmentation (EI-MS) of this peak including prominent fragment ions at m/z 302, 260, 201, 145, 129, 103, and 85 identical with those found for alditol acetates derivatives of QuiNAc std. The right box insert shows the predicted primary and secondary MS fragments of C1-deuterated alditol-acetate of derived QuiNAc. (TIFF) [file pone.0133790.s001.tiff]

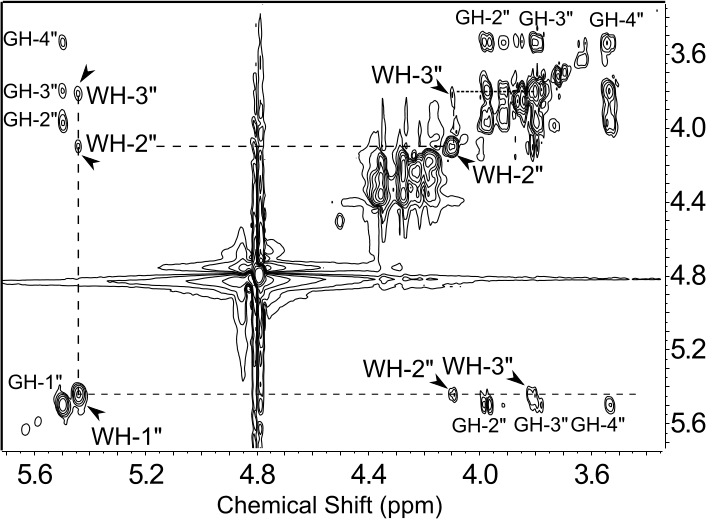

Supplement: S2 Fig — (TIFF) [file pone.0133790.s002.tiff]
